# Epidemiology of childhood injuries in Saudi Arabia: a scoping review

**DOI:** 10.1186/s12887-021-02886-8

**Published:** 2021-09-25

**Authors:** Hadeel Albedewi, Nouf Al-Saud, Abdulhameed Kashkary, Ada Al-Qunaibet, Salem M. AlBalawi, Suliman Alghnam

**Affiliations:** 1 Department of Community Health Sciences, College of Applied Medical Sciences, King Saud University, Riyadh, Saudi Arabia; 2 Biostatistics, Epidemiology and Scientific Computing Department, King Faisal Specialist Hospital and Research Center, Riyadh, Saudi Arabia; 3 Saudi Public Health Authority, Riyadh, Saudi Arabia; 4 Population Health Section-King Abdullah International Medical Research Center, King Saud bin Abdulaziz University for Health Sciences, Riyadh, Saudi Arabia

**Keywords:** Unintentional injuries, Fractures, Burns, Road traffic, Poisoning, Oral injuries

## Abstract

**Background:**

Injury is the leading cause of death among Saudi children. Despite that, much remains unknown on the epidemiology and the extent of burden. This scoping review aims to describe previous literature on injury burden, including types, causes, and outcomes.

**Methods:**

We conducted a scoping literature search of English published articles on injuries among Saudi children between 0 to 18 years old using Scopus, MEDLINE, and Web of Science between January 2000 and December 2020. The primary outcome was the type and the cause of childhood injuries. Data extraction was based on specified data elements that included study characteristics and epidemiological parameters. The STROBE checklist was used to assess the quality of publications.

**Results:**

The initial review identified 3,384 studies. Of which, 36 studies met the inclusion criteria. A total of 20,136 children were included; of them, 69% were males. Among studies that examined overall injuries, falls represented 31.9%, while 25.1% were due to Motor Vehicle Collision (MVC). The leading cause of fractures was falls (37.9%), followed by MVC (21.5%). The leading cause was flames (52.1%) followed by scald (36.4%) for burns. While for poisoning, medications were the leading cause of (39.9%), followed by toxic household products (25.7%). Weighted mortality rates were 5.2% for overall injuries, 8.3% for fractures of the skull and spine, and 17.4% for burns.

**Conclusions:**

MVC and falls are associated with the highest share of injuries in the kingdom. These findings can guide prevention efforts to reduce injury burden and improve population health. Further population-based research is warranted to explore the determinants of childhood injuries across all regions of Saudi Arabia.

**Supplementary Information:**

The online version contains supplementary material available at 10.1186/s12887-021-02886-8.

## Background

Injuries are the leading cause of morbidity and mortality worldwide [1]. According to the Global Burden of Disease (GBD) study, 973 million individuals suffer from injuries that require medical attention, and around 4.8 million die every year [2]. Childhood injuries are of particular concern. In 2011, more than 630,000 children died because of injuries [3]. In the United States, unintentional injuries were the leading cause of death in 2019 [4]. According to the Centers for Disease Control and Prevention (CDC), the leading cause of death from unintentional injuries was MVC followed by drowning, burns, and suffocation, whereas unintentional fall remains the most common cause for nonfatal injuries.

The burden of injuries is not limited to preventable deaths. Millions of children require hospitalization, and some endure lifelong disabilities that could negatively affect their stages of development. According to a report by the World Health Organization (WHO), loss of Disability-Adjusted Life Years (DALYs) worldwide is predicted to be primarily caused by MVC by 2030 [5]. Around 16.6% of loss DALYs among children in Saudi Arabia are due to injuries and of which 8.1% are due to MVC [6].

Injuries are not random events, but they are often associated with predictable factors. Age is associated with a higher risk of serious injury [7]. A previous systematic review suggested a bimodal distribution peaking at the two opposite age extremities [7]. Teenagers between 15 to 19 have an increased risk due to increased exposure to hazards and risk-taking behaviours. On the other hand, young infants are also at a high risk of injuries [7]. Gender also plays a role in the risk of fatal and nonfatal injuries among children [7]. Although males are at a higher risk for mortality due to all types of injury, the male to female ratio varies by the cause of injury. For example, motorcycle crashes, falls, and firearm-related deaths are ten times higher among males, while pedestrian-related deaths are only slightly greater among males [7, 8].

Child injuries are a public health concern for both high-income and low-income countries but more pronounced in countries undergoing extreme urbanization and industrialization [9, 10]. However, the risk of injury-related deaths is higher among low- and middle-income than high-income countries [11]. Children of low- and middle-income countries are exposed to more hostile environments; open fires, unstable construction sites, unprotected stairways, absence of safe play space, and lack of safe storage of chemicals [12]. Poverty is a risk factor for unintentional injuries even within high-income countries [13].

In Saudi Arabia, two injuries were ranked among the ten leading causes of death: transport injuries and unintentional injuries, according to the GBD [14]. The majority of preventable deaths in Saudi children (82.5%) were attributed to injuries [15]. The leading cause for injury mortality was MVC at 60.6% and was most commonly found among 13-18-year-olds, followed by drowning at 13.4% and most commonly found among 6-12-year-olds [15].

Approximately 31% of the Saudi population are children and adolescents [14]. Therefore, the consequences of injuries will have direct effects on population health. Despite that, limited and fragmented exploration of the burden of childhood injuries may negatively impact prevention. Further understanding of the epidemiology of child injuries will facilitate efforts in prevention and guide future research to understand the magnitude of the problem. Therefore, we aim in this review to describe the magnitude of childhood injuries, including the most common types and causes across gender and age groups in Saudi Arabia.

## Methods

### Search strategy and study selection:

A literature search was performed in January 2021 using Scopus, MEDLINE, and Web of Science for any study published in English between January 2000 and December 2020. We used search limits for source type, document type, year, language, country, and age. The search strategy for each database is detailed in Supplementary file 1. Additional records were identified from reference lists of selected articles, Saudi digital library, and search engines. One reviewer (H.A) reviewed titles and abstracts to assess relevance and collected relevant titles and abstracts for full text assessment. Deduplication was performed using Microsoft Excel. Full text manuscripts were reviewed and evaluated against the eligibility criteria.

### Eligibility criteria

**Fig. 1:**
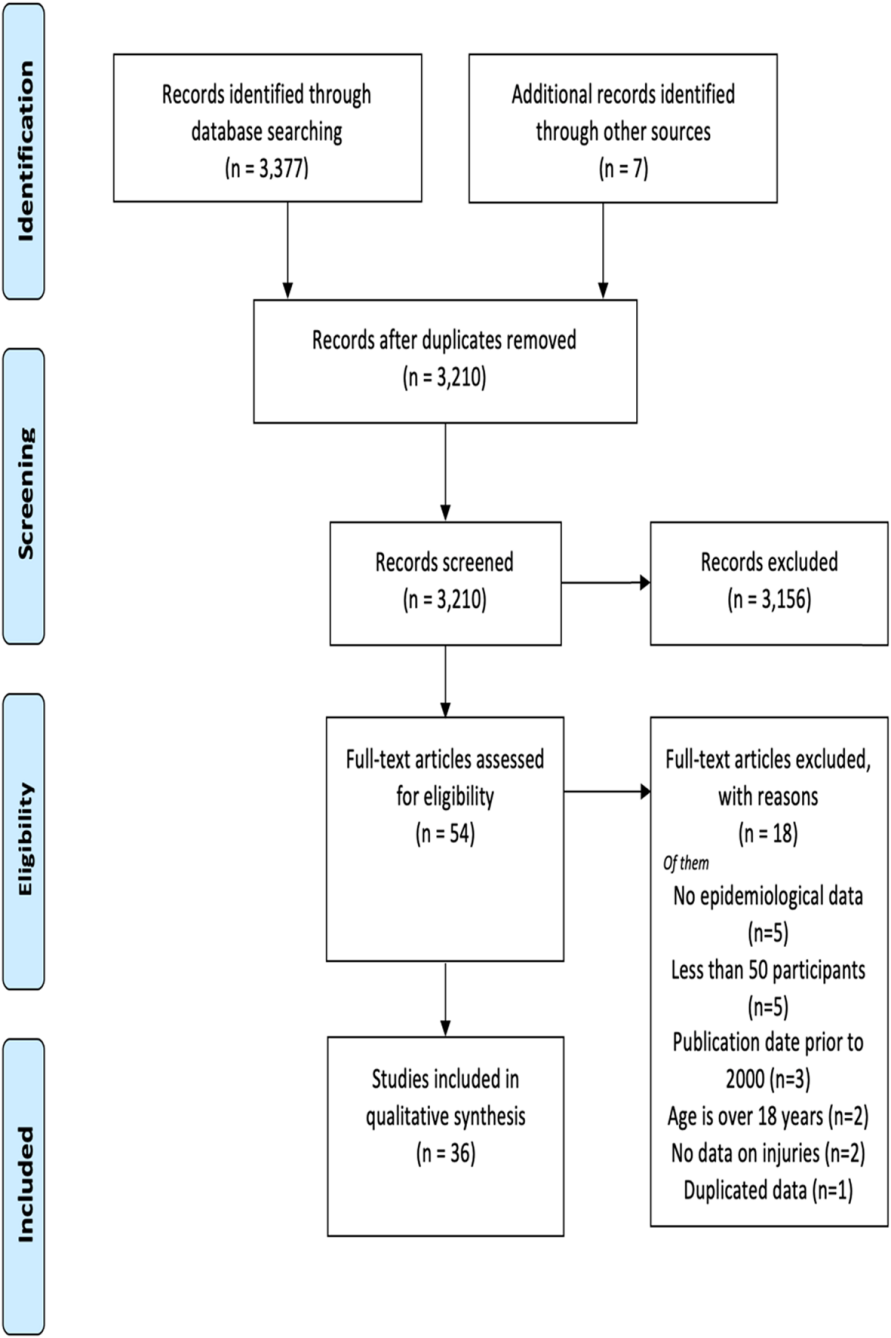
PRISMA flow diagram of articles screening and selection

The databases were searched from April to May 2021 to identify articles published between January 1st, 2000, to December 31st, 2020. The timeframe was chosen because medical documentation and research in Saudi Arabia became mature only in the last two decades. The steps we followed for study selection are shown in (Fig. 1). To be included in this review, a study had to meet the following criteria:

1. Children (between 0 to 18 years of age.).
2. The publication date is between 2000 and 2020.
3. Published in English.
4. Contain epidemiological data such as age and gender.
5. Focused on injuries including drowning, fall, trauma, fracture, burn, MVC, poisoning, and suffocation.

Exclusion criteria were:

1)Reviews, case studies, or abstracts.

2)Sample size of fewer than 50 children.

### Type of studies

The type of studies included was retrospective and prospective cohorts in addition to cross-sectional studies.

### Data extraction and synthesis

The primary outcome was the type and the cause of childhood injuries and their distribution among age groups, gender, and regions. The number of children, year of publication, study timeframe, study type, region, age, gender, type and cause of injury, and mortality rate were recorded by one reviewer (H.A). No authors have been contacted to obtain any additional data. After reviewing the literature, studies were categorized based on the type of injury into six categories: overall injuries, fractures, burns, MVC injuries, oral injuries, and poisoning. Weighted percentages were calculated for gender, causes, and mechanism of injury in addition to the reported overall mortality rate in each category.

### Quality assessment

The quality of studies was assessed by two independent reviewers (HA and NA) using the Strengthening the Reporting of Observational Studies in Epidemiology (STROBE) statement [16]. The STROBE checklist includes 22 items distributed as 1 for abstract, 2 for introduction, 9 for the methods, 5 for the results, and 4 for the discussion, and 1 for funding. Disagreements were resolved by consensus.

## Results

The initial search identified 3,384 studies. After screening titles and abstracts, 3,330 publications were excluded for being irrelevant or duplicates. The full text of 54 studies was assessed for eligibility, where 18 did not meet the inclusion criteria and were excluded. Thirty-six studies have met the inclusion criteria and were included in this scoping review (Fig. 1). In total, 20,136 participants were included. Of the total population, 13,890 were males (69%), and 5,596 were females (27.8%), while gender was not reported for 650 children (3.2%). Twenty-one studies were conducted in the central region - Riyadh, which accounts for the majority (58%) of the studies. As for the remaining studies, five were conducted in the eastern region, four, three, and one were conducted in the western region, Southern region, and Northern region, respectively. Two studies were conducted in several regions of the country.

**Fig. 2:**
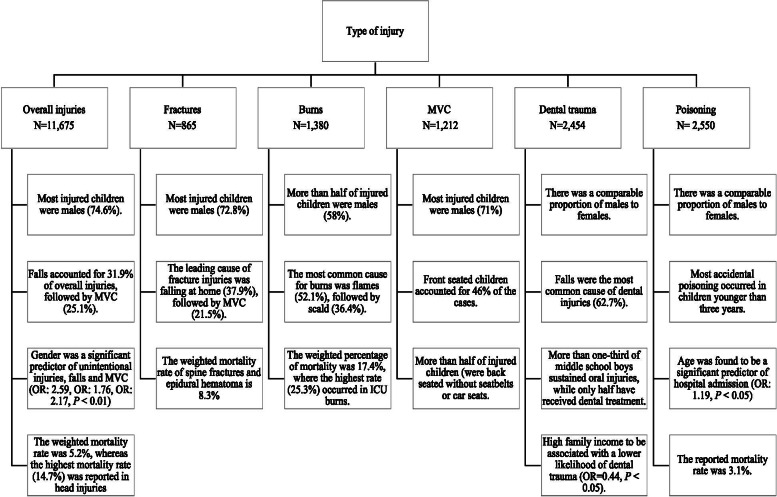
Summary of main study findings on childhood injuries in Saudi Arabia

Of the included publications, eleven studies explored overall injuries. Five studies focused on fractures, five on burns, four on MVC injuries, four on oral injuries, and seven examined poisoning. A visual summary of some of the studies’ findings is presented in Fig. 2. Based on the STROBE checklist, the quality of studies ranged from 5 to 19, with the majority scores being between 13 and 16 (Supplementary file 2). The most common missing items were the explanation of the study size, variables, handling quantitative variables, controlling for bias, and generalizability.

### Overall injuries

Eleven thousand six hundred seventy-five participants were included in the overall injuries’ category (Table 1) [17–27]. Most injured children were males (74.6%). In fact, gender was found to be a significant predictor of unintentional injuries, falls and MVC with higher odds for males (OR: 2.59, OR: 1.76, OR: 2.17, respectively, *P* < 0.01) [17, 26]. According to a household survey conducted among 1,650 participants, 22% of children sustained an injury in the past year [17]. A more recent study with a smaller sample (n=283) suggested that the prevalence of unintentional childhood injuries was 24.7% in the past 12 months [26]. The weighted percentage of falls accounted for 31.9% of overall injuries, followed by MVC representing 25.1%. Head injuries were most likely to be associated with MVC followed by falls [18]. Furthermore, blunt injuries caused by falls and MVC were the leading cause of deaths in overall injuries [21]. Additionally, MVC was reported to be the primary cause of injury that led to an extended length of stay [22]. In fact, the mechanism of injury was found to be a significant predictor of extended length of stay with higher odds for MVC and burns (OR: 16.2, OR: 14.5, respectively, *P* < 0.001) as opposed to falls [22].

**Table 1:** Study characteristics and relevant findings of studies of childhood overall injuries

Author	Timeframe	Study type (sample size)	Relevant findings	Region	STROBE Score
(Gad et al., 2011) [17]	2011	Cross-sectional N=1650	History of injury in the past 12 months was reported by 22.2% of the children. Injuries were higher among males compared to females (26% vs. 18%, respectively, *P* < 0.001). Male gender was a significant predictor of falls and MVC (OR: 1.76, OR: 2.17, respectively, *P* < 0.001) The most common type of injury fall (40.4%) followed by MVC (15%) and food poisoning (8.8%). The reported mortality rate of injured children was 1.5%	Riyadh	17
(Alhabdan et al., 2013) [18]	2001 - 2009	Retrospective N=1219	The most common cause of injury was MVC (34.2%) followed by pedestrian injury (30.3%) and falls (28.4%). Most of head injuries (66.3%) occurred in children younger than 12 years. The overall mortality rate was 14.7%.	Riyadh	14
(Alanzi, 2013) [19]	2010 - 2011	Retrospective N=200	The prevalence of fingertip injury was significantly higher among males compared to females ((59.5% vs. 40.5%, *P<*0.001). Children younger than 5 years were more likely to have fingertip incidents. The most common cause for fingertip injury was house doors.	Riyadh	13
(Assiry, 2014) [20]	2012 - 2014	Retrospective N=71	Head injuries were most likely to occur in males (76.1%). The major cause was MVC (63.4%) followed by falls (32.4%).	Southern region - Asir	13
(Alnasser et al., 2018) [21]	2009 - 2014	Retrospective N=1762	Males represented 68.4% of admitted children following injuries. The most common type of injury was blunt trauma (73%), followed by burns (17%) and penetrating injuries (10%). Blunt trauma was mainly caused by MVC (50%) followed by fall (40%). The leading cause of deaths among patients was blunt injury (92%). The mortality rate was 2.8%	Riyadh	16
(Alghnam et al., 2019) [22]	2001 - 2018	Retrospective N=5563	Males represented 75.8% of admitted children following injuries. The most common type of injury was fall (31.5%), followed by an MVC (28.5%) The prevalence of children who had extended length of stay was 14%. The major cause of injury that led to extended length of stay was MVC. Mechanism of injury was a significant predictor of extended length of stay with higher odds for MVC and burns (OR: 16.2, OR: 14.5, respectively, *P* < 0.001) as opposed to falls.	Riyadh	19
(Al-Qurashi et al., 2019) [23]	2005 - 2015	Retrospective N=51	Males represented 66.7% of the drowning incidents. Brain death occurred in 4% of the cases, 2% had severe neurological issues and 94% fully recovered. Most cases (74.5%) occurred in the sea (56.9%) at the night with no lifeguard present (92.2%).	Eastern Region -Dammam	15
(Alzamil et al., 2019) [24]	2018	Cross-sectional N=323	Visual loss was present in 39% of Pediatric Ophthalmology Clinic (N=818) whereas 22.9% were blind. Trauma was the most common cause for unilateral blindness (20.7%) followed by refractive errors (15.5%).	Eastern region - Dhahran	15
(AlAteeq et al., 2020) [25]	2016 - 2017	Cross-sectional N=491	Most injuries occurred in males (64%). The leading cause of injuries was fall (47.7%) followed by hot liquids and chemical exposure (14.5%). The most common injury types were fractures, dislocations, and subluxations (47.3%) followed by penetration injuries (21%) and burn injuries (17.5%). The mortality rate was 1.2%	Riyadh	15
(Alkhamis & Abdulkader, 2020) [26]	2020	Cross-sectional N=283	The prevalence of unintentional childhood injuries was 24.7% in the past 12 months. The prevalence of unintentional childhood injuries was significantly higher in males compared to females (32.5% vs. 15.9%, respectively, *P=*0.001). Male gender was a significant predictor of unintentional injuries (OR: 2.59, *P=*0.003). Most injuries occurred at home (74.3%). The most common cause of injurie was falls (62.9%) followed by burns (22.9%).	Riyadh	18
(Al-Sarheed et al., 2020) [27]	2005 - 2018	Retrospective N=62	Most trauma injuries occurred in males (77.4%). The most common cause of injury was MVC (59.7%), followed by pedestrian accidents (21%) and falls (14.5%). The mortality rate was 12.7%	Riyadh	15

*MVC* Motor Vehicle Collision, *OR* Odds ratio

Another critical childhood injury was drowning. A study showed that males represented 66.7% of the drowning incidents. Among this population, drowning led to brain death (4%), and 2% of victims sustained severe neurological damage [23]. The study, which was conducted in the eastern region, reported that most cases occurred in the sea (74.5%) at night (56.9%) with no lifeguard present (92.2%).

Fingertip injuries were identified as one mechanism and were significantly higher among males (59.5%) compared to females [19]. These injuries were commonly caused by house doors and more prevalent among children younger than five years. Overall, the mortality rate for overall injuries was only reported in five studies. Among those studies, the weighted mortality rate was 5.2%, whereas the highest mortality rate (14.7%) was reported in head injuries [18].

### Fractures

Fracture injuries studies included 865 participants (Table 2) [28–32]. The majority of reported fractures occurred among males (72.8%). The ratio of male to female was 2.68:1. The leading cause of fracture injuries was falling at home (37.9%), followed by MVC (21.5%). Other causes for fractures included door slams, direct hits, and pedestrian injuries. Fractures occurred mainly among older children as close half of children were between 13-18 of age [30]. At the same time, epidural hematoma was more likely to occur among children between 5-14 years of age than other children [29, 31]. On the other hand, nonaccidental fractures were more common in preschoolers, followed by infants [32]. The mortality rate was reported in two studies conducted on spine fractures and epidural hematoma, both with a rate of 8.3% [29, 31].

**Table 2:** Study characteristics and relevant findings of studies of childhood fractures

Author	Timeframe	Study type (sample size)	Relevant findings	Region	STROBE Score
(Alomran et al., 2012) [28]	2004 - 2009	Retrospective N=254	Most fractures occurred in males (62.6%). Almost half of injuries (48.8%) occurred at home. The major cause of injury was fall at home (48.8%) followed by motor vehicle accidents (29.52%)	Riyadh	13
(Al-Habib et al., 2014) [29]	2001 - 2009	Retrospective N=120	The majority of injured children were males (83.3%) (*P=*0.003). The major cause of spine injury was MVC (60.8%) followed by pedestrian injuries (20.8%), fall-related injuries (15%). Traumatic spine fractures were more common in children age between 16-18 years. The mortality rate was 8.3%.	Riyadh	16
(Al-Jasser et al., 2015) [30]	2005 - 211	Retrospective N=361	The majority of fractures occurred in males (80.6%) (*P<*0.0001). Injured children were most likely (46.2%) to be between 13-18 of age. The major cause of injury was fall at home (35.7%) followed by door slam (25%) and sport related (17.1%).	Riyadh	16
(Umerani et al., 2018) [31]	2012 - 2014	Retrospective N=72	Epidural hematoma was most likely to occur in males (65.3%) and in age between 5-14 years. The most common cause for epidural hematoma was MVC (52.8%) followed by falls (34.7%). The mortality rate was 8.3%.	Eastern region - Dammam	11
(Jawadi et al., 2019) [31]	2009 - 2015	Retrospective N=58	Nonaccidental fractures were most likely to occur in males (59%) and preschoolers (45%) followed by infants (34%). The rate of hospital admission was 70%. Physical abuse accounted for 44.6% of the incidents whilst 51.8% was due to neglect. The most common type of injury was fall (54.8%) followed by direct hit (26.1%).	Riyadh	16

### Burns

Burn studies included 1,380 children (Table 3) [33–37]. Of those, 58% were males. The extent of burns was minor in most cases (less than 20% of Total Body Surface Area (TBSA)). The most common cause for burns was flames (52.1%), followed by scald (36.4%). Two studies showed that most burns (60%) occurred among toddlers and preschoolers [33, 34]. About half of the victims admitted to the Intensive Care Unit (ICU) were between 5-10 years of age [36]. Four studies have reported mortality rates; the weighted percentage of mortality was 17.4%, where the highest rate (25.3%) occurred among those admitted to the ICU.

**Table 3:** Study characteristics and relevant findings of studies of childhood burns

Author	Timeframe	Study type (sample size)	Relevant findings	Region	STROBE Score
(Priyadarshini & Kumar, 2015) [33]	2007 - 2011	Retrospective N=100	Most burns occurred in children younger than 6 years of age (59%). Comparable proportions of male and female injured children (51 vs. 49%, respectively). 51% had minor burns (0-20% TBSA). Cause of burn was 47% scald and 42% flame. Overall mortality: 15%	Northern region - Sakaka, Al Jouf,	12
(Alharthy et al., 2016) [33]	2013	Retrospective N= 148	More than half of burn injuries (54%) occurred in male children. The mean TBSA was 5%. Most of burns occurred in toddlers and preschoolers (80%). Scald burns were the most common type of burn (76.4%) followed by flame (15.5%). No reported deaths.	Riyadh	16
(Alturki et al., 2019) [35]	2011 - 2016	Retrospective N=95	More than half of injured children were males (56.8%). Most burn cases were infants, toddlers, and preschoolers (72.5%). Scald burn was the most common type of burn (68.8%).	Western region - Jeddah	16
(Akkam et al., 2020) [36]	2009 - 2018	Retrospective N=787	Slightly higher male victims than females (56.4 vs. 43.6%). Most injuries (73.8%) occurred at home. Almost half of subjects 52% had minor burn (10-19% TBSA) The main cause of injury was flame (81%). Overall mortality: 25%	Riyadh	17
(Mater et al., 2020) [37]	2016 - 2017	Retrospective N=250	Higher proportion of burns among males compared to females (68% vs. 32%, respectively). The mean TBSA was 16.54% Scald was the major cause of burn (63.4%). Overall mortality: 4.8%	Riyadh	12

*TBSA* Total Body Surface Area

### MVC

One thousand two hundred twelve children were included in the MVC studies (Table 4) [38–41]. Overall, males represented the majority (71%) of injured victims. One study showed that most injuries (71%) were sustained by pedestrians, followed by passengers (27%) [38]. Front seated children accounted for 46% of the cases with a higher rate of isolated head, neck, or facial injuries than back seated children (51.2% vs. 25%, *P* = 0.01) [39]. On the other hand, children seated in the back had higher rates of rollover (52.1% vs 24.4%, *P* = 0.02), ejection (41.7% vs 22%, *P* = 0.05), and occupant death ratio (14.8% vs 4%, *P* = 0.04) and were more likely to have long bone or pelvic fractures (60.4% vs 36.6%, *P* = 0.02) [39]. Moreover, a study showed that 38.3% of children injured in MVC sustained head injuries, whereas facial injuries were sustained by 34.8% of children [41]. According to that study, 2.4% lost their lives due to the MVC. More than half of injured children (53.8%) were back seated without seatbelts or car seats, while 9.1% were driving [41].

**Table 4:** Study characteristics and relevant findings of studies of childhood MVC injuries

Author	Timeframe	Study type (sample size)	Relevant findings	Region	STROBE Score
(Crankson, 2006) [38]	1994 - 2003	Retrospective N=664	Males represented 71% of MVC injuries MVC injuries represented 42% of pediatric trauma admissions. Mechanism of injury was 71% pedestrians, 27% auto passengers, 1.5% bicyclists and 0.5% motorcyclists.	Riyadh	12
(Al-Jazaeri et al., 2012) [39]	2001 - 2010	Retrospective N=89	Boys represented 72% of MVC injuries Injured children were front seated in 46% of the cases. Front seated children had higher rate of isolated head, neck or facial injuries (51.2% vs 25%, *P*=.01) Back seated children had higher rates of rollover (52.1% vs 24.4%, *P*=.02), ejection (41.7% vs 22%, P=.05), and occupant death ratio (14.8% vs 4%, *P*=.04) and were more likely to have long bone or pelvic fractures (60.4% vs 36.6%, *P*=.025).	Riyadh	9
(Mohmmedthani et al., 2018) [40]	2011 - 2016	Retrospective N=206	Most of the cases (72.5%) were younger than 10 years. Males represented 75.9% of MVC injures. The isolated femoral shaft fractures represented 70.9%, while femoral shaft fractures with associated injuries represented 29.1%.	Western region - Medina	15
(Alghnam et al., 2020) [41]	2016 - 2019	Retrospective N=253	The proportion of head injury in following MVC was 38.3% and facial injury was 34.8%. Male represented 68.8% of the study population. The majority of injured children (53.8%) were back seated without seatbelts or safety seats while 9.1% were driving. The mortality rate was 2.4%.	Several regions	18

*MVC* Motor Vehicle Collision

### Oral and dental trauma

The number of patients included in oral and dental trauma studies was 2,454 (Table 5) [42–45]. There was a comparable proportion of males to females [43, 44]. While the other two were conducted solely in males [42, 45]. Falls were the most common cause of dental injuries (62.7%) [43, 44]. More than one-third (39.5%) of middle school boys sustained oral injuries, while only half have received dental treatment [45]. The same study showed high family income to be associated with a lower likelihood of dental trauma (OR=0.44, *P* < 0.05) [45].

**Table 5:** Study characteristics and relevant findings of studies of childhood dental trauma

Author	Timeframe	Study type (sample size)	Relevant findings	Region	STROBE Score
(Al-Majed et al., 2001) [42]	1997	Cross-sectional N=1216 Gender: male	The prevalence of dental trauma was 32.8% among boys aging between 5-6 years and 34.3% among those aged 12-14 years.	Riyadh	9
(Al-Malik, 2009) [43]	2005-2006	Cross-sectional N=112	The prevalence of traumatic oral injury was higher among males (70.5%, *P* < 0.05), and age between 9-11 years Fall was the most common cause of traumatic oral injuries (68%) Injuries where most likely to occur in the street in males (69.6%) and at home (60.6%) in females.	Western region - Jeddah	13
(Gupta et al., 2018) [44]	2016	Cross-sectional N=868	The prevalence of traumatic dental injury was 9.79% with fall being the most common cause (62.4%) Traumatic dental injuries were more common among male with a male female ratio of 1.6:1.	Southern region - Jazan	13
(Al-Ansari & Nazir, 2020) [45]	2020	Cross-sectional N=258 Gender: male	As per a self-reported questionnaire, 39.5% of middle school male children had experienced dental trauma whilst only 20.5% received treatment. Higher family income was associated with lower likelihood of dental trauma (OR: 0.44, *P* < 0.05) and lower odds of receiving treatment for dental trauma (OR: 0.41, *P* < 0.05).	Eastern region Dammam - Alkhobar	17

*OR* Odds ratio

### Poisoning and toxicological exposure

Studies on childhood poisoning and toxicological exposure included 2,550 children (Table 6) [46–52]. Most of the accidental poisoning occurred among children younger than three years. Further, age was found to be a significant predictor of hospital admission (OR: 1.19, P < 0.05) [48]. The male-female ratio was comparable (1.17:1). The most common mechanism was ingestion with drugs associated with 39.9% of the cases, followed by toxic household products (25.7%). One study reported that no action was taken by parents (90.2%) following the incidents [47]. A study conducted in several regions of the kingdom reported a 3.1% mortality rate [51].

**Table 6:** Study characteristics and relevant findings of studies of childhood poisoning and toxicological exposure

Author	Timeframe	Study type (sample size)	Relevant findings	Region	STROBE Score
(Izuora & Adeoye, 2001) [46]	1992 - 1998	Retrospective N= 168	The majority of accidental poisoning (63%) occurred in children aged between 1-3 years. The most frequently involved substance was drugs (64.3%). Most cases (>60%) were asymptomatic. No reported deaths.	Eastern region - Hafr Albatin	12
(Al-Binali et al., 2009) [47]	1990 - 1995	Retrospective N=72	The most common type of corrosives was 5.25% hypochlorite (50%), followed by kerosene in (16.7%), and caustic soda (12.5%). No action was taken by parents of 90.2% of the cases. No reported deaths.	Southern region - Asir	10
(Alanazi et al., 2015) [48]	2009 - 2011	Retrospective N=315	The majority of child poison occurred among toddlers (72%). Most poisoning incidents occurred in males (59%). The most frequently involved substance was drugs (63.2%). Age was a significant predictor of hospital admission (OR: 1.19, *P* < 0.05).	Riyadh	17
(Bakhaidar et al., 2015) [49]	2008 - 2012	Retrospective N=129	Children under 12 years represented 44.2% chemical poisoning admissions. More than half of poisoning cases (54.3%) were females. Accidental poisoning occurred in 92.9% of children younger than 12 years. No reported deaths.	Western region	15
(Alghadeer et al., 2018) [50]	2010 - 2016	Retrospective N=735	The mean age was 2.7 years. The most common type of poisoning was drugs (70%). The majority of cases were asymptomatic.	Riyadh	16
(Alanazi et al., 2018) [51]	2018	Cross-sectional N=96	More than third (35%) of poisoning cases were between 4-8 years and 24% were younger than 4 years. Poisoning injury were most common in males 57.3% compared to 42.7%. Accidental poisoning occurred in 94.8% whereas 5.2% were intended. The most common cause of poisoning was spoiled food (55.2%) The mortality rate was 3.1%.	Several regions	5
(Alruwaili et al., 2019) [52]	2016 - 2017	Cross-sectional N=1035	Most of toxicological exposed children (78.7%) were asymptomatic and 47.8% did not need an intervention. Most exposure incidents (91%) occurred in children younger than 6 years with 62.2% of them being younger than 3 years. The most frequently involved substance class was toxic household products in children younger than 6 years and pesticides in children 6 years or older.	Riyadh	15

*OR* Odds ratio

## Discussion

This scoping review suggests that falls and MVC are the leading causes of injuries in the kingdom. Similarly, a global study conducted in low-and middle-income countries found fall and MVC to be the most common mechanism of childhood injuries [53].

Many MVC injuries are preventable using evidence-based safety measures such as seatbelts, car seats, or helmets. However, they remain a neglected focus in the kingdom. In this respect, a study conducted in 2018 to assess the use of child restraint systems found that only one-third of the families reported having child restraint systems while only half of them were consistently using them [54]. Additionally, more than half of the families reported setting children on the passenger’s lap [54]. Therefore, further investment in public health interventions to reduce falls and MVC is warranted to reduce their burden on population health.

Our findings show that males represented most overall injuries, fractures, burns, MVC, and dental trauma. These results are consistent with the public data obtained from the CDC, stating that males accounted for 58.4% of unintentional injuries that occurred between 2001 and 2019 in the United States [55]. Likewise, in low- and middle-income countries, male children were more susceptible to injuries than females [53]. This gender disparity can be attributed to the greater risk-taking tendency in males [56]. Thus, gender-specific prevention strategies may help reduce the burden of associated injuries [57].

Although the mortality rate was relatively low, it may reflect the underlying resources for capturing injury data. However, the rate was exceptionally high among more critical cases such as ICU burns (25.3%) and head injuries (14.7%), followed by fractures (8.3%). Correspondingly, a study on childhood mortality in the eastern region showed that half of the deaths (51%) were attributed to accidents, with male children representing 69% of the cases [58]. In addition, the autopsy has revealed that head injury was the leading cause of death (27%) among the autopsied cases [58]. These are high rates, and national actions need to be undertaken to reduce them.

Primary prevention of injuries provides the best value and return on the outcome. The Child Safety Action Plan (CSAP) in European Union serves as an excellent example for tackling mortality resulting from childhood injuries [59]. This policy was first developed through assessing the situation with government engagement, followed by setting a vision, goals, and objectives, and finally implementing the best evidence-based practices that the government endorsed.

Our findings have some policy and public health implications, namely supporting the Saudi 2030 vision [60]. One of the pillars of this Vision is to extend life expectancy from 74 to 80 and improve quality of life. Our research describes the magnitude of the problem and highlights the need for intersectoral interventions. As the Vision focuses on preventing health risks, preventive interventions of childhood injuries, namely fall, and MVC need to be implemented. One of the Vision’s objectives is to promote traffic safety which could potentially aid in MVC prevention. Another implication is highlighting the need for investing in secondary prevention by dealing with injuries once they occur to reduce their consequences. A recent cross-sectional study found a low level of awareness about first aid to childhood injuries such as burns, drowning, and choking among 39% of Saudi parents [61]. However, most parents (78%) were willing to take a first aid class. This reinforces the need to implement sustainable awareness-raising strategies at the family level.

This is the first scoping review of the epidemiology of childhood injuries in Saudi Arabia to the best of our knowledge. However, we acknowledge that our review has several limitations. First, the lack of socioeconomic data may not reflect the variation of the prevalence and the mechanisms of injuries. Second, we were also unable to capture and generalize the age groups due to the missing continuous data and the inconsistency in age group classification across the studies. Third, most studies were retrospective chart review studies which can be more susceptible to bias. There is a significant need for population-based and longitudinal studies to provide more substantial evidence of the burden of injuries on population health of the kingdom. Fourth, except for one study, the primary focus of the included publications was unintentional injuries, which emphasizes the need to extend the current research in Saudi Arabia to nonaccidental childhood injuries. Finally, there was a substantial difference in publication rates across the regions of Saudi Arabia as more than half of the included studies were conducted in the central region-Riyadh, which affects the generalizability of our findings. This can be attributed to the presence of highly equipped trauma centers and research institutions in the region. Clearly, there is a disparity in our ability to capture the true burden in other regions accurately. Therefore, it is crucial to conduct further studies on the prevalence and magnitude of childhood injuries across all regions of Saudi Arabia while maintaining a high-quality research and reporting practices.

## Conclusions

This review found that the leading causes and mechanisms of childhood injuries were falls and MVC. Unfortunately, all types of injuries resulted in substantial mortality rates, and there is a definite need for national action to be undertaken to reduce them. Overall, further research should be carried out to capture the determinants of childhood injuries across all regions of Saudi Arabia.

## Supplementary Information

**Additional file 1: Supplementary Table 1**. Search terms and search limits for all databases.

**Additional file 2: Supplementary Table 2**. The STROBE assessment of all included studies.

## Data Availability

The study data is available from the corresponding author upon request.

## Abbreviations

CDC: Centers for Disease Control and Prevention
CSAP: Child Safety Action Plan
DALY: Disability-adjusted life years
GBD: Global Burden of Disease
ICU: Intensive Care Unit
MVC: Motor Vehicle Collision
STROBE: Strengthening the Reporting of Observational Studies in Epidemiology
TBSA: Total Body Surface Area
WHO: World Health Organization

## References

[1] Bustos Córdova E, Cabrales Martínez RG, Cerón Rodríguez M, Naranjo López MY (2014). Epidemiología de lesiones no intencionales en niños: revisión de estadísticas internacionales y nacionales. Bol Med Hosp Infant Mex.

[2] Haagsma JA, Graetz N, Bolliger I, Naghavi M, Higashi H, Mullany EC (2016). The global burden of injury: incidence, mortality, disability-adjusted life years and time trends from the Global Burden of Disease study 2013. Inj Prev.

[3] World Health Organization: Child Injuries. Available from: https://www.who.int/violence_injury_prevention/child/injury/en/.

[4] Centers for Disease Control and Prevention. Web-based Injury Statistics Query and Reporting System (WISQARS) [Online]. Fatal Injury Reports, Centers for Disease Control and Prevention. 2003. Available from: 2003. Available from: www.cdc.gov/injury/wisqars. 2021 Mar 13.

[5] Peden M OK, Ozanne-smith J, Hyder AA., Branche C, Rahman FA, et al. Geneva: World Health Organization; 2008. WHO Guidelines Approved by the Guidelines Review Committee. 2008.

[6] Murray CJL, Aravkin AY, Zheng P, Abbafati C, Abbas KM, Abbasi-Kangevari M (2020). Global burden of 87 risk factors in 204 countries and territories, 1990–2019: a systematic analysis for the Global Burden of Disease Study 2019. Lancet.

[7] Dowd MD, Keenan HT, Bratton SL (2002). Epidemiology and prevention of childhood injuries. Crit Care Med.

[8] Baker SP, O'Neill B, Ginsburg MJ, Li G (1992). The Injury Fact Book. Second Edition.

[9] Mock CN, Abantanga F, Cummings P, Koepsell TD (1999). Incidence and outcome of injury in Ghana: a community-based survey. Bull World Health Organ.

[10] Smith GS, Barss P (1991). Unintentional injuries in developing countries: the epidemiology of a neglected problem. Epidemiol Rev.

[11] Tupetz A, Friedman K, Zhao D, Liao H, Isenburg MV, Keating EM (2020). Prevention of childhood unintentional injuries in low- and middle-income countries: A systematic review. PLoS One.

[12] Sethi D, Aldridge E, Rakovac I, Makhija A. Worsening Inequalities in Child Injury Deaths in the WHO European Region. Int J Environ Res Public Health. 2017;14(10):1128. 10.3390/ijerph14101128 PMC5664629 28954422

[13] Lao Z, Gifford M, Dalal K (2012). Economic cost of childhood unintentional injuries. Int J Prev Med.

[14] Tyrovolas S, El Bcheraoui C, Alghnam SA, Alhabib KF, Almadi MAH, Al-Raddadi RM (2020). The burden of disease in Saudi Arabia 1990–2017: results from the Global Burden of Disease Study 2017. Lancet Planetary Health.

[15] Almuneef M, Saleheen H, AlBuhairan F, Al-Eissa M, Al Muntaser M, Al Alem H, et al. Child mortality in Saudi Arabia: Time for action at all levels. Int J Pediatrics Adolescent Med. 2020. 10.1016/j.ijpam.2020.06.003. 10.1016/j.ijpam.2020.06.003 PMC8319650 34350329

[16] von Elm E, Altman DG, Egger M, Pocock SJ, Gotzsche PC, Vandenbroucke JP (2008). The Strengthening the Reporting of Observational Studies in Epidemiology (STROBE) statement: guidelines for reporting observational studies. J Clin Epidemiol.

[17] Gad A, AL-Eid R, Al-Ansary S, Saeed A b, Kabbash A (2011). Pattern of injuries among children and adolescents in Riyadh, Saudi Arabia: a household survey. J Trop Pediatr.

[18] Alhabdan S, Zamakhshary M, AlNaimi M, Mandora H, Alhamdan M, Al-Bedah K (2013). Epidemiology of traumatic head injury in children and adolescents in a major trauma center in Saudi Arabia: implications for injury prevention. Ann Saudi Med.

[19] Alanzi A. Fingertip injuries in paediatric patients — experiences at an emergency centre in Saudi Arabia. J Pak Med Assoc. 2013;63(6):675–9. 23901663

[20] Assiry K, Abdulmutali H, Alqahtani A, Alyahya A, Elawad M (2014). Traumatic Head Injuries in Children: Experience from Asir.

[21] Alnasser A, Othman A, Mobaireek O, Alharthy N, Aljerian N, et al. Epidemiology of pediatric trauma at a tertiary hospital in Riyadh, Saudi Arabia. J Nat Sci Biol Med. 2018;9(2):247–51.

[22] Alghnam S, Towhari JA, Al Babtain I, Al Nahdi M, Aldebasi MH, Alyami M (2019). The associations between injury mechanism and extended hospital stay among pediatric patients: findings from a trauma Center in Saudi Arabia. BMC Pediatr.

[23] Al-Qurashi FO, Yousef AA, Aljoudi A, Alzahrani SM, Al-Jawder NY, Al-Ahmar AK (2019). A Review of Nonfatal Drowning in the Pediatric-Age Group: A 10-Year Experience at a University Hospital in Saudi Arabia. Pediatr Emerg Care.

[24] Alzamil WM, Alshamlan FT, Alkhaldi HM, Almubaiyd AM, Alsaif AA, Alhamad JR (2019). Causes of blindness in a pediatric age group at a tertiary healthcare center in the eastern province of Saudi Arabia. Saudi Med J.

[25] AlAteeq MA, Alsulayhim AK, AlHargan F, AlSamaani IS, Alyousef M, AlDossari A (2020). Morbidity Patterns of Non-Traffic Unintentional Injuries Among the Pediatric Age Group Attending the Emergency Department at King Abdul-Aziz Medical City, Riyadh, Saudi Arabia. Cureus.

[26] Alkhamis KN, Abdulkader RS (2020). Assessment of unintentional childhood injuries and associated factors in the pediatric clinics of a tertiary care hospital in Riyadh, Saudi Arabia. J Family Commun Med.

[27] Al-Sarheed S, Alwatban J, Alkhaibary A, Babgi Y, Al-Mohamadi W, Masuadi EM (2020). Cervical spine clearance in unconscious pediatric trauma patients: a level l trauma center experience. Childs Nerv Syst.

[28] Alomran A, Bubshait D, Sadat-Ali M. Epidemiology of Pediatric Fractures and Dislocations: Analysis of In-Patients. Bahrain Med Bull. 2012;34(4).

[29] Al-Habib A, Alaqeel A, Marwa I, Almohammadi M, Al Shalaan H, AlEissa S (2014). Causes and patterns of spine trauma in children and adolescents in Saudi Arabia: implications for injury prevention. Ann Saudi Med.

[30] Al-Jasser FS, Mandil AM, Al-Nafissi AM, Al-Ghamdi HA, Al-Qattan MM (2015). Epidemiology of pediatric hand fractures presenting to a university hospital in Central Saudi Arabia. Saudi Med J.

[31] Umerani MS, Abbas A, Aziz F, Shahid R, Ali F, Rizvi RK (2018). Pediatric Extradural Hematoma: Clinical Assessment Using King's Outcome Scale for Childhood Head Injury. Asian J Neurosurg.

[32] Jawadi AH, Benmeakel M, Alkathiri M, Almuneef MA, Philip W, Almuntaser M (2019). Characteristics of Nonaccidental Fractures in Abused Children in Riyadh, Saudi Arabia. Saudi J Med Med Sci.

[33] Priyadarshini S, Kumar P. Epidemiology and profile of pediatric burns- a retrospective review. Soc Wound Care Res. 2015;8:29–33.

[34] Alharthy N, Al Mutairi M, AlQueflie S, Nefesa AB, Manie NB, Nafesa SB (2016). Pattern of burns identified in the Pediatrics Emergency Department at King Abdul-Aziz Medical City: Riyadh. J Nat Sci Biol Med.

[35] Alturki N, Alkahtani M, Daghistani M, Alyafi T, Khairy S, Ashi M (2019). Incidence and risk factors for deep vein thrombosis among pediatric burn patients. Burns..

[36] Akkam AY, Joarder A, Cruz-Marcelino N, Mitra B, Alshehri S, Almazroua F (2020). Epidemiology of pediatric patients admitted to a burns ICU in Saudi Arabia. Burns Open.

[37] Mater ME, Yamani AE, Aljuffri AA, Binladen SA (2020). Epidemiology of burn-related infections in the largest burn unit in Saudi Arabia. Saudi Med J.

[38] Crankson SJ (2006). Motor vehicle injuries in childhood: a hospital-based study in Saudi Arabia. Pediatr Surg Int.

[39] Al-Jazaeri A, Zamakhshary M, Al-Omair A, Al-Haddab Y, Al-Jarallah O, Al-Qahtani R (2012). The role of seating position in determining the injury pattern among unrestrained children involved in motor vehicle collisions presenting to a level I trauma center. Ann Saudi Med.

[40] Mohmmedthani T, Alfraidi T, Sonbol A, Almulla A, Hetaimish B, Alrashidi Y. Prevalence of femoral shaft fracture and associated injuries among children after road traffic accidents in a Saudi Arabian trauma center. J Musculoskeletal Surg Res. 2018;2:66–9.

[41] Alghnam S, Jastaniah E, Alwahaibi W, Albabtain IT, Alqublan S, Bajwaiber M (2020). The prevalence of head and facial injuries among children in Saudi Arabia following road traffic crashes. Ann Saudi Med.

[42] Al-Majed I, Murray JJ, Maguire A (2001). Prevalence of dental trauma in 5-6- and 12-14-year-old boys in Riyadh, Saudi Arabia. Dent Traumatol.

[43] Al-Malik M (2009). Oral injuries in children attending a hospital in Saudi Arabia. J Maxillofac Oral Surg.

[44] Gupta M, Apathsakayan R, Lnu A (2018). Traumatic Injuries to the Anterior Teeth among Children of Jazan, Kingdom of Saudi Arabia: A Screening Study. World J Dentistry.

[45] Al-Ansari A, Nazir M (2020). Prevalence of Dental Trauma and Receipt of Its Treatment among Male School Children in the Eastern Province of Saudi Arabia. Sci World J.

[46] Izuora GI, Adeoye A (2001). A seven-year review of accidental poisoning in children at a Military Hospital in Hafr Al Batin, Saudi Arabia. Ann Saudi Med.

[47] Al-Binali AM, Al-Shehri MA, Abdelmoneim I, Shomrani AS, Al-Fifi SH (2009). Pattern of corrosive ingestion in southwestern Saudi Arabia. Saudi J Gastroenterol.

[48] Alanazi M, Al-jerisay M, Al-assiri M, Afeesh L, Alhammad F, Salam M. Hospital Performance Indicators and Their Associated Factors in Acute Child Poisoning at a Single Poison Center, Central Saudi Arabia. Medicine. 2015;94(25):e2399. 10.1097/MD.0000000000002339 PMC5291612 26717371

[49] Bakhaidar M, Jan S, Farahat F, Attar A, Alsaywid B, Abuznadah W (2015). Pattern of drug overdose and chemical poisoning among patients attending an emergency department, western Saudi Arabia. J Community Health.

[50] Alghadeer S, Alrohaimi M, Althiban A, Kalagi NA, Balkhi B, Khan AA (2018). The patterns of children poisoning cases in community teaching hospital in Riyadh, Saudi Arabia. Saudi Pharm J.

[51] Alanazi M, Alshammari T, Alshehri M, Alaradi A, Alshammari T, Alsagre S, et al. Futures and the outcome of treatment of poisoned children and adolescents admitted to emergency units in different areas of Saudi Arabia. Egypt J Hospital Med. 2018;73(2):5970–975.

[52] Alruwaili ND, Halimeh B, Al-Omar M, Alhatali B, Sabie II, Alsaqoub M (2019). An epidemiological snapshot of toxicological exposure in children 12 years of age and younger in Riyadh. Ann Saudi Med.

[53] He S, Lunnen JC, Puvanachandra P, Amar S, Zia N, Hyder AA (2014). Global childhood unintentional injury study: multisite surveillance data. Am J Public Health.

[54] Alsanea M, Masuadi E, Hazwani T (2018). Use of child restraint system and patterns of child transportation in Riyadh, Saudi Arabia. PLoS One.

[55] Centers for Disease Control and Prevention. Web-based Injury Statistics Query and Reporting System (WISQARS) [Online]. (2003). Nonfatal Injury Reports, Centers for Disease Control and Prevention (producer). Available from: 2003 [Available from: www.cdc.gov/injury/wisqars. 2021 Mar 13].

[56] Byrnes JP, Miller DC, Schafer WD (1999). Gender differences in risk taking: A meta-analysis. Psychol Bull.

[57] Sorenson SB (2011). Gender disparities in injury mortality: consistent, persistent, and larger than you'd think. Am J Public Health.

[58] Alsaif DM, Almadani OM, Almoghannam S, Hamdi D, Al-Farayedhi MA, Kharosha MA (2018). Child Fatalities in Dammam: A Call for Child Fatality Reviews in Arab Countries. J Forensic Sci.

[59] MacKey M, Vincenten J (2007). Action Planning for Child Safety: A Strategic and Coordinated Approach to Reducing the Number One Cause of Death for Children in Europe.

[60] Vision2030.gov.sa. 2021. National Transformation Program | Saudi Vision 2030. [online] Available at: https://www.vision2030.gov.sa/en/programs/NTP [Accessed 14 March 2021].

[61] Habeeb KA, Alarfaj G (2020). Saudi parents awareness regarding burn, choking, and drowning first aid in children. J Family Med Prim Care.

