# Supplementary material for: Epidemiology of childhood injuries in Saudi Arabia: a scoping review
Source: BMC Pediatr. 2021 Sep 25;21:424. doi: 10.1186/s12887-021-02886-8 (PMC8464152; doi:10.1186/s12887-021-02886-8)
Supplement: Supplementary file 1 — Additional file 1: Supplementary Table 1. Search terms and search limits for all databases. [file 12887_2021_2886_MOESM1_ESM.docx]

Supplementary Table 1. Search terms and search limits for all databases

| Database | Search terms | Search limits |
| --- | --- | --- |
| Scopus | ("injury" OR "fracture" OR "burn" OR "trauma" OR "drowning" OR "motor vehicle collision" OR "poisoning" OR "suffocation") AND "Saudi Arabia" AND ("pediatric" OR "children") (all fields) | Year: 2000 to 2020  Language: English  Source type: Journal  Document type: Article  Country: Saudi Arabia |
| MEDLINE | injury OR fracture OR burn OR trauma OR motor vehicle collision OR drowning OR poisoning OR suffocation (all fields) AND Saudi Arabia (country) | Publication date: 2000 to 2020  Language: English  Source type: Academic journal  Age: All child (0-18 years). |
| Web of Science | "injury" OR "fracture" OR "burn" OR "trauma" OR "drowning" OR "motor vehicle collision" OR "poisoning" OR "suffocation" (title) AND "Saudi Arabia" (all fields) AND "children" (all fields) | Publication date: 2000 to 2020  Language: English  Document type: Article  Country: Saudi Arabia |
