# Supplementary material for: Epidemiology of childhood injuries in Saudi Arabia: a scoping review
Source: BMC Pediatr. 2021 Sep 25;21:424. doi: 10.1186/s12887-021-02886-8 (PMC8464152; doi:10.1186/s12887-021-02886-8)
Supplement: Supplementary file 2 — Additional file 2: Supplementary Table 2. The STROBE assessment of all included studies. [file 12887_2021_2886_MOESM2_ESM.docx]

The STROBE assessment of included publications.

Supplementary Table 2.The STROBE assessment of included studies 17-25.

|  | **Item No** | **17** | **18** | **19** | **20** | **21** | **22** | **23** | **24** | **25** |
| --- | --- | --- | --- | --- | --- | --- | --- | --- | --- | --- |
| **Title and abstract** | 1 | 1 | 1 | 1 | 1 | 1 | 1 | 1 | 1 | 1 |
| Background/rationale | 2 | 1 | 1 | 1 | 1 | 1 | 1 | 1 | 0 | 1 |
| Objectives | 3 | 1 | 1 | 1 | 0 | 1 | 1 | 1 | 1 | 1 |
| Study design | 4 | 1 | 1 | 1 | 1 | 1 | 1 | 1 | 1 | 1 |
| Setting | 5 | 1 | 1 | 1 | 1 | 1 | 1 | 1 | 1 | 1 |
| Participants | 6 | 1 | 1 | 1 | 1 | 1 | 1 | 1 | 1 | 1 |
| Variables | 7 | 0 | 0 | 1 | 1 | 1 | 1 | 1 | 1 | 1 |
| Data sources/ measurement | 8* | 1 | 1 | 1 | 1 | 1 | 1 | 1 | 1 | 1 |
| Bias | 9 | 0 | 0 | 0 | 0 | 0 | 1 | 0 | 0 | 0 |
| Study size | 10 | 1 | 0 | 0 | 0 | 0 | 0 | 0 | 0 | 0 |
| Quantitative variables | 11 | 0 | 0 | 0 | 0 | 0 | 0 | 0 | 0 | 0 |
| Statistical methods | 12 | 1 | 1 | 0 | 0 | 0 | 1 | 1 | 1 | 1 |
| Participants | 13* | 1 | 1 | 0 | 1 | 1 | 1 | 1 | 0 | 1 |
| Descriptive data | 14* | 1 | 1 | 1 | 1 | 1 | 1 | 1 | 1 | 1 |
| Outcome data | 15* | 1 | 1 | 1 | 1 | 1 | 1 | 1 | 1 | 1 |
| Main results | 16 | 1 | 1 | 1 | 1 | 1 | 1 | 1 | 1 | 1 |
| Other analyses | 17 | 0 | 0 | 0 | 0 | 0 | 0 | 0 | 0 | 0 |
| Key results | 18 | 1 | 0 | 1 | 1 | 1 | 1 | 1 | 1 | 1 |
| Limitations | 19 | 1 | 1 | 0 | 0 | 1 | 1 | 0 | 1 | 0 |
| Interpretation | 20 | 1 | 1 | 1 | 1 | 1 | 1 | 1 | 1 | 1 |
| Generalisability | 21 | 0 | 0 | 0 | 0 | 0 | 1 | 0 | 0 | 0 |
| Funding | 22 | 1 | 0 | 0 | 0 | 1 | 1 | 0 | 1 | 0 |
| Total | 22 | 17 | 14 | 13 | 13 | 16 | 19 | 15 | 15 | 15 |

Supplementary Table 3. The STROBE assessment of included studies 26-34.

|  | **Item No** | **26** | **27** | **28** | **29** | **30** | **31** | **32** | **33** | **34** |
| --- | --- | --- | --- | --- | --- | --- | --- | --- | --- | --- |
| **Title and abstract** | 1 | 1 | 1 | 1 | 1 | 1 | 1 | 1 | 1 | 1 |
| Background/rationale | 2 | 1 | 1 | 0 | 1 | 1 | 0 | 1 | 0 | 1 |
| Objectives | 3 | 1 | 1 | 1 | 1 | 1 | 0 | 1 | 0 | 1 |
| Study design | 4 | 1 | 1 | 1 | 1 | 1 | 1 | 1 | 1 | 1 |
| Setting | 5 | 1 | 1 | 1 | 1 | 1 | 1 | 1 | 1 | 1 |
| Participants | 6 | 1 | 1 | 1 | 1 | 1 | 0 | 1 | 1 | 1 |
| Variables | 7 | 0 | 1 | 1 | 1 |  | 1 | 1 | 1 | 0 |
| Data sources/ measurement | 8* | 1 | 1 | 0 | 1 | 1 | 1 | 1 | 0 | 1 |
| Bias | 9 | 0 | 0 | 0 | 0 | 0 | 0 | 0 | 0 | 0 |
| Study size | 10 | 1 | 0 | 0 | 0 | 0 | 0 | 0 | 0 | 0 |
| Quantitative variables | 11 | 0 | 0 | 0 | 0 | 0 | 0 | 0 | 0 | 1 |
| Statistical methods | 12 | 1 | 1 | 0 | 1 | 1 | 0 | 0 | 0 | 1 |
| Participants | 13* | 1 | 1 | 1 | 1 | 1 | 1 | 1 | 1 | 0 |
| Descriptive data | 14* | 1 | 1 | 1 | 1 | 1 | 1 | 1 | 1 | 1 |
| Outcome data | 15* | 1 | 1 | 1 | 1 | 1 | 1 | 1 | 1 | 1 |
| Main results | 16 | 1 | 1 | 1 | 1 | 1 | 1 | 1 | 1 | 0 |
| Other analyses | 17 | 0 | 0 | 0 | 0 | 0 | 0 | 0 | 0 | 0 |
| Key results | 18 | 1 | 0 | 1 | 1 | 1 | 1 | 1 | 1 | 1 |
| Limitations | 19 | 1 | 1 | 1 | 1 | 1 | 0 | 1 | 0 | 1 |
| Interpretation | 20 | 1 | 0 | 1 | 1 | 1 | 1 | 1 | 1 | 1 |
| Generalisability | 21 | 1 | 1 | 0 | 0 | 0 | 0 | 0 | 0 | 1 |
| Funding | 22 | 1 | 0 | 0 | 0 | 1 | 0 | 1 | 1 | 1 |
| Total | 22 | 18 | 15 | 13 | 16 | 16 | 11 | 16 | 12 | 16 |

Supplementary Table 4. The STROBE assessment of included studies 35-43.

|  | **Item No** | **35** | **36** | **37** | **38** | **39** | **40** | **41** | **42** | **43** |
| --- | --- | --- | --- | --- | --- | --- | --- | --- | --- | --- |
| **Title and abstract** | 1 | 1 | 1 | 1 | 1 | 1 | 1 | 1 | 0 | 1 |
| Background/rationale | 2 | 1 | 1 | 1 | 1 | 1 | 1 | 1 | 1 | 1 |
| Objectives | 3 | 0 | 1 | 1 | 0 | 0 | 1 | 1 | 1 | 1 |
| Study design | 4 | 1 | 1 | 1 | 0 | 0 | 1 | 1 | 0 | 0 |
| Setting | 5 | 1 | 1 | 1 | 1 | 0 | 1 | 1 | 1 | 1 |
| Participants | 6 | 1 | 1 | 1 | 1 | 1 | 1 | 1 | 0 | 1 |
| Variables | 7 | 1 | 1 | 0 | 1 | 1 | 0 | 1 | 1 | 1 |
| Data sources/ measurement | 8* | 1 | 1 | 0 | 1 | 1 | 1 | 1 | 1 | 1 |
| Bias | 9 | 0 | 0 | 0 | 0 | 0 | 0 | 0 | 0 | 0 |
| Study size | 10 | 0 | 0 | 0 | 0 | 0 | 0 | 0 | 0 | 0 |
| Quantitative variables | 11 | 1 | 0 | 1 | 0 | 1 | 0 | 0 | 0 | 0 |
| Statistical methods | 12 | 1 | 1 | 1 | 0 | 1 | 1 | 1 | 0 | 1 |
| Participants | 13* | 1 | 1 | 0 | 0 | 1 | 0 | 1 | 1 | 1 |
| Descriptive data | 14* | 1 | 1 | 0 | 0 | 0 | 1 | 1 | 1 | 0 |
| Outcome data | 15* | 1 | 1 | 1 | 1 | 1 | 1 | 1 | 1 | 1 |
| Main results | 16 | 1 | 1 | 0 | 1 | 0 | 1 | 1 | 1 | 0 |
| Other analyses | 17 | 0 | 0 | 0 | 0 | 0 | 0 | 0 | 0 | 1 |
| Key results | 18 | 1 | 1 | 1 | 1 | 0 | 1 | 1 | 0 | 1 |
| Limitations | 19 | 1 | 1 | 1 | 1 | 1 | 1 | 1 | 0 | 0 |
| Interpretation | 20 | 1 | 1 | 1 | 1 | 0 | 1 | 1 | 0 | 1 |
| Generalisability | 21 | 0 | 1 | 0 | 1 | 0 | 0 | 1 | 0 | 0 |
| Funding | 22 | 0 | 0 | 1 | 0 | 0 | 1 | 1 | 0 | 0 |
| Total | 22 | 16 | 17 | 12 | 12 | 9 | 15 | 18 | 9 | 13 |

Supplementary Table 5. The STROBE assessment of included studies 44-52.

|  | **Item No** | **44** | **45** | **46** | **47** | **48** | **49** | **50** | **51** | **52** |
| --- | --- | --- | --- | --- | --- | --- | --- | --- | --- | --- |
| **Title and abstract** | 1 | 1 | 1 | 1 | 1 | 1 | 1 | 1 | 0 | 1 |
| Background/rationale | 2 | 1 | 1 | 1 | 1 | 1 | 1 | 1 | 0 | 1 |
| Objectives | 3 | 1 | 1 | 1 | 1 | 1 | 1 | 1 | 0 | 1 |
| Study design | 4 | 0 | 1 | 1 | 1 | 1 | 1 | 1 | 1 | 1 |
| Setting | 5 | 1 | 0 | 1 | 1 | 1 | 1 | 1 | 0 | 1 |
| Participants | 6 | 1 | 1 | 0 | 0 | 1 | 0 | 1 | 0 | 0 |
| Variables | 7 | 0 | 1 | 0 | 1 | 1 | 1 | 1 | 0 | 1 |
| Data sources/ measurement | 8* | 1 | 1 | 1 | 1 | 1 | 1 | 1 | 1 | 1 |
| Bias | 9 | 0 | 0 | 0 | 0 | 0 | 0 | 0 | 0 | 0 |
| Study size | 10 | 0 | 1 | 0 | 0 | 0 | 0 | 0 | 0 | 0 |
| Quantitative variables | 11 | 0 | 1 | 0 | 0 | 1 | 1 | 0 | 0 | 0 |
| Statistical methods | 12 | 1 | 1 | 0 | 0 | 1 | 1 | 1 | 0 | 0 |
| Participants | 13* | 0 | 0 | 1 | 0 | 1 | 1 | 1 | 0 | 0 |
| Descriptive data | 14* | 1 | 1 | 1 | 1 | 0 | 1 | 1 | 1 | 1 |
| Outcome data | 15* | 1 | 1 | 1 | 1 | 1 | 1 | 1 | 1 | 1 |
| Main results | 16 | 0 | 1 | 1 | 0 | 1 | 0 | 1 | 0 | 1 |
| Other analyses | 17 | 0 | 0 | 0 | 0 | 0 | 0 | 0 | 0 | 0 |
| Key results | 18 | 1 | 1 | 1 | 1 | 1 | 1 | 1 | 1 | 1 |
| Limitations | 19 | 1 | 1 | 0 | 0 | 1 | 1 | 1 | 0 | 1 |
| Interpretation | 20 | 1 | 1 | 1 | 0 | 1 | 1 | 1 | 0 | 1 |
| Generalisability | 21 | 0 | 1 | 0 | 0 | 0 | 0 | 0 | 0 | 1 |
| Funding | 22 | 1 | 0 | 0 | 0 | 1 | 0 | 0 | 0 | 1 |
| Total | 22 | 13 | 17 | 12 | 10 | 17 | 15 | 16 | 5 | 15 |
